# Highly sensitive droplet digital PCR for detection of *RET* fusion in papillary thyroid cancer

**DOI:** 10.1186/s12885-023-10852-z

**Published:** 2023-04-20

**Authors:** Mengke Chen, Junyu Xue, Ye Sang, Wenting Jiang, Weiman He, Shubin Hong, Weiming Lv, Haipeng Xiao, Rengyun Liu

**Affiliations:** 1 Institute of Precision Medicine, The First Affiliated Hospital, Sun Yat-Sen University, No. 58, Zhongshan Second Road, Guangzhou, 510080 China; 2 Department of Endocrinology, The First Affiliated Hospital, Sun Yat-Sen University, No. 58, Zhongshan Second Road, Guangzhou, 510080 China; 3 Department of Breast and Thyroid Surgery, The First Affiliated Hospital, Sun Yat-Sen University, No. 58, Zhongshan Second Road, Guangzhou, 510080 China

**Keywords:** *RET* fusion, ddPCR, Molecular diagnosis, Thyroid cancer

## Abstract

**Background:**

Thyroid cancer is the most frequent malignancy of the endocrine system, of which papillary thyroid cancer (PTC) is the predominant form with a rapid increasing incidence worldwide. *Rearranged during transfection* (*RET*) fusions are common genetic drivers of PTC and the potent RET inhibitor selpercatinib has been recently approved for treating advanced or metastatic *RET* fusion-positive thyroid cancer. In this study we aimed to develop a droplet digital PCR (ddPCR) system to accurately detect *RET* fusion in PTC samples.

**Methods:**

The frequency and distribution of *RET* fusions in PTC were analyzed using genomic data of 402 PTC patients in The Cancer Genome Atlas (TCGA) database. To establish the ddPCR system for detecting *CCDC6*::*RET* fusion, a plasmid containing *CCDC6*::*RET* infusion fragment was constructed as standard template, the annealing temperature and concentrations of primers and probe were optimized. The analytical performance of ddPCR and quantitative reverse transcription PCR (qRT-PCR) were assessed in standard templates and tissue samples from 112 PTC patients. Sanger sequencing was performed in all the *RET* fusion-positive samples identified by ddPCR.

**Results:**

*RET* fusions were observed in 25 (6.2%) of the 402 TCGA samples, and 15 (60%) of the RET fusion-positive patients had the *CCDC6*::*RET* fusion. Compared with qRT-PCR, the ddPCR method showed a lower limit of detection (128.0 and 430.7 copies/reaction for ddPCR and qRT-PCR, respectively). When applying the two methods to 112 tissue samples of PTC, eleven (9.8%) *CCDC6*::*RET* fusion-positive samples were detected by qRT-PCR, while ddPCR identified 4 additional positive samples (15/112, 13.4%). All the *CCDC6*::*RET* fusion-positive cases identified by ddPCR were confirmed by Sanger sequencing except for one case with 0.14 copies/uL of the fusion.

**Conclusion:**

The accurate and sensitive ddPCR method reported here is powerful to detection *CCDC6*::*RET* fusion in PTC samples, application of this method would benefit more *RET* fusion-positive patients in the clinic.

**Supplementary Information:**

The online version contains supplementary material available at 10.1186/s12885-023-10852-z.

## Background

Thyroid cancer is the most common type of endocrine cancer, with an increasing overall incidence in recent decades [1]. Based on the type of cells from which the cancer grows, thyroid cancer is generally divided into two categories: follicular cell-derived cancers, including papillary thyroid cancer (PTC), follicular thyroid cancer (FTC), poorly differentiated thyroid cancer (PDTC) and anaplastic thyroid cancer (ATC); and parafollicular C cell-derived medullary thyroid cancer (MTC). The two categories of thyroid cancers have different genetic background. Specifically, over half of the follicular cell-derived thyroid cancers are driven by *BRAF* V600E, *TERT* promoter mutations, and/or genetic alterations in the PI3K/AKT pathway, while the major genetic driver of MTC is germline or somatic rearranged during transfection (*RET*) mutations [2–5].

Interestingly, although *RET* mutation is rarely observed in follicular cell-derived thyroid cancers, *RET* fusion occurs frequently in PTC and PDTC [6, 7], particular in the patients with young age and environmental radiation exposure [8–12]. The most common breakpoint of *RET* was observed in intron 11, and then it fused with coiled-coil domain containing 6 (*CCDC6*), nuclear receptor co-activator 4 (*NCOA4*), or other N-terminal partner genes [13]. These rearrangements lead to constitutively ligand-independent RET tyrosine kinase domain (TKD) activation and act as oncogenic drivers in cancer progression [14].

Major advanced were made recently in the field of targeted therapy for *RET*-altered cancers [15]. Based on efficacy data from clinical trials, two highly selective RET inhibitors selpercatinib and pralsetinib were approved by the FDA in the year 2020 for treating patients with metastatic *RET* fusion-positive non-small cell lung cancer (NSCLC), advanced or metastatic *RET*-mutant MTC and advanced or metastatic *RET* fusion-positive thyroid cancer [16–19]. To catch the right patients for prescribing selpercatinib or pralsetinib in the clinic, the first essential step is accurate detection of *RET* mutation and fusions. Compared with conventional methods used for gene mutation or fusion detection, the droplet digital PCR (ddPCR) showed several advantages, including high sensitivity and accuracy [20, 21]. The ddPCR for *RET* mutation detection has been well established [22, 23], but there is no report on how to detect *RET* fusions by ddPCR. Herein, in this study we developed a ddPCR method for *RET* fusion detection and compared its performance with qRT-PCR in clinical samples from 112 PTC patients.

## Methods

### Patients

The Cancer Genome Atlas (TCGA) database for PTC patients was downloaded, and the distribution of key driver genetic alterations and the frequency of *RET* fusion subtypes were analyzed in 402 patients with whole exome sequencing data [6]. A total of 112 patients (87 women and 25 men), with a median (interquartile range) age of 36 (33–39) years, who were diagnosed and treated for PTC at The First Affiliated Hospital of Sun Yat-sen University between 2017 and 2019, were enrolled for *RET* fusion detection. This study was approved by the ethics committee of our hospital and informed patient consent to participate in this study was obtained where required.

### RNA extraction and cDNA preparation

The total RNA from each tissue was extracted by TRIzol™ Reagent (cat#15,596,018, Invitrogen, Waltham, MA, USA) according to the user guide. Then 1 µg of isolated RNA was used to generate first strand cDNA using a RevertAid First Strand cDNA Synthesis Kit (cat#K1622, Thermo Fisher Scientific, Waltham, MA, USA). 1ug RNA, 1 μl of Oligo(dT)_18_ primer and nuclease-free water were mixed gently to a total volume of 12 μl. To reduce the influence of GC-rich or secondary structures of RNA, RNA solution was incubated at 65 °C for 5 min and chilled on ice. Then 2 μl of 10 mM dNTP mix, 4 μl of 5 × reaction buffer, 1 μl of RiboLock RNase inhibitor, 1 μl of RevertAid RT was added to each tube. This mixture was incubated at 42 °C for 60 min and at 70 °C for 5 min. Followed, the product of the first strand cDNA synthesis was diluted ten times with nuclease-free water (final concentration 5 ng/ul) then stored at − 80 °C until it was used.

### Standard template construction

A plasmid containing *CCDC6* (Exon 1)::*RET* (Exon 12) infusion fragment was constructed and linearized as the standard template to evaluate the performance of qRT-PCR and ddPCR. Synthetic DNA sequence was inserted into pUC57 vector. The plasmid was linearized with restriction endonuclease NotI (NEB, R3189S) and XhoI (NEB, R0146S), and frozen at − 80 °C. The gene copy number was estimated by calculation formula: copies/ul = con.(ng/ul)*(10^–9^)*(6.02*10^23^) / (DNA length*660) [24].

### ddPCR

The forward primer (5’- TGCAGCAAGAGAACAAGGTG -3’), reverse primer (5’- TGACCACTTTTCCAAATTCGCC-3’), and probe (5’-FAM- ATTCCCTCGGAAGAACTTG -MGB-3’) were purified with high-performance liquid chromatography (HPLC). Optimized reactions were performed in 20 ul of duplex ddPCR reaction mix that consisted of 1X Droplet PCR Supermix (cat#186–3024, Bio-Rad, München, Germany), forward and reverse primers (final concentration of 800 nmol/L for each primer), probe (final concentration of 200 nmol/L) and 1 ul of template cDNA. After well mixed, the mixture was partitioned into 20,000 nanoliter-sized water-in-oil droplets by QX200™ Droplet Generator (cat#1,864,002, Bio-Rad). After gently transferred to 96-well plate and sealed, the PCR reaction was carried out in a Thermocycler T100 (Bio-Rad) using the following program: 95 °C for 5 min, 40 cycles of 94 °C for 30 s and 62.5 °C for 60 s (ramp rate: 2.5 °C/sec), 1 cycle of 98 °C for 10 min and holding at 12 °C. Droplets were counted at room temperature using the QX200 Droplet Reader (Cat#1,864,003, Bio-Rad) and analyzed using the Quantasoft software. The total number of droplets detected by each reaction was equal or exceed 10,000.

### qRT-PCR assay

The primers, probe, and cDNA used for qRT-PCR were same as the ddPCR. The reaction was performed using TaqMan® Fast Advanced Master Mix (#4,444,557, Applied Biosystems) and by the Applied Biosystems QuantStudio 5 Real-Time PCR System under the following program: preincubated at 50 °C for 10 min and 95 °C for 2 min; followed 40 cycles of 95 °C for 10 s and 60 °C for 30 s. The results were analyzed by the statistical analysis system of the instrument.

### PCR and Sanger sequencing

The PCR reaction was performed using OneTaq Hot Start DNA Polymerase (#M0481S, NEB) on the Applied Biosystems ProFlex PCR System under the following program: preincubated at 94 °C for 30 s; followed 45 cycles of 94 °C for 20 s, 60 °C for 30 s and 68 °C for 30 s; final extension at 68 °C for 10 min. The PCR products were separated by electrophoresis in a 2% agarose gel and recognized by Sanger sequencing.

### Statistical analysis

The Oncoprinter from cBioPortal (https://www.cbioportal.org/oncoprinter) was used to analyze and visualize the genetic alterations profiling [25]. χ^2^ test or Fisher’s exact test were selected for comparing differences between categorical variables by IBM SPSS (version 26.0). GraphPad Prism (version 7.0) was used to do the linear regression. And Probit regression analysis for LoD was done by MedCalc software (Version 20.121).

## Results

### Distribution of RET fusions in PTC

**Fig. 1:**
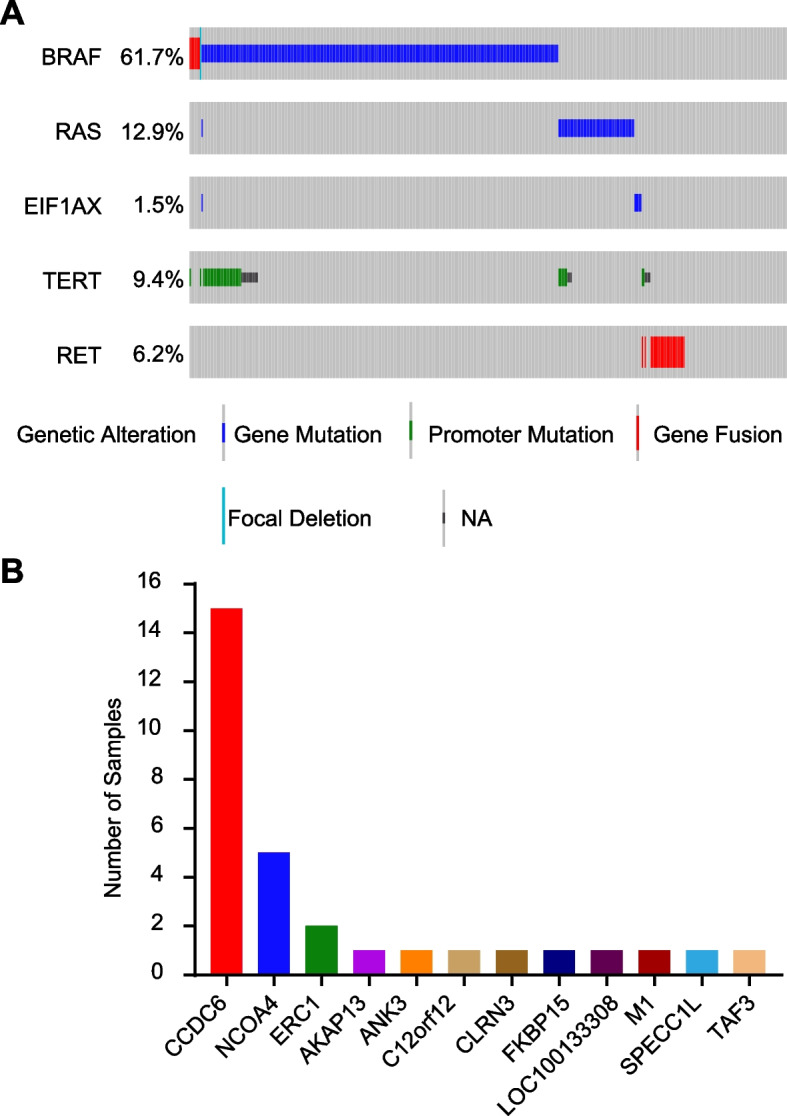
Genetic alterations of selected genes in PTC. **A** Distribution of common driver genes in 402 PTC patients from the TCGA dataset. **B** Frequency of different *RET* fusion subtypes in PTCs

Among the 402 PTC patients with adequate sequencing data for genomic analysis, the RET fusions were observed in 25 (6.2%) samples. They were mutually exclusive with other driver mutations or fusions, including *BRAF*, *RAS* and *EIF1AX*, and the majority of the *RET* fusion-positive samples (24 of 25) occurred in patients that did not harbor *TERT* promoter mutations (Fig. 1A). As shown in Fig. 1B, the most frequent type of RET fusions in PTCs was *CCDC6*::*RET* (also named *RET*-*PTC1*), accounting for 60% (15 of 25) of *RET* fusion-positive samples and for 3.7% (15 of 402) of all PTCs. Therefore, we next focused on *CCDC6*::*RET* detection.

#### Development of ddPCR system for CCDC6::RET detection

**Fig. 2:**
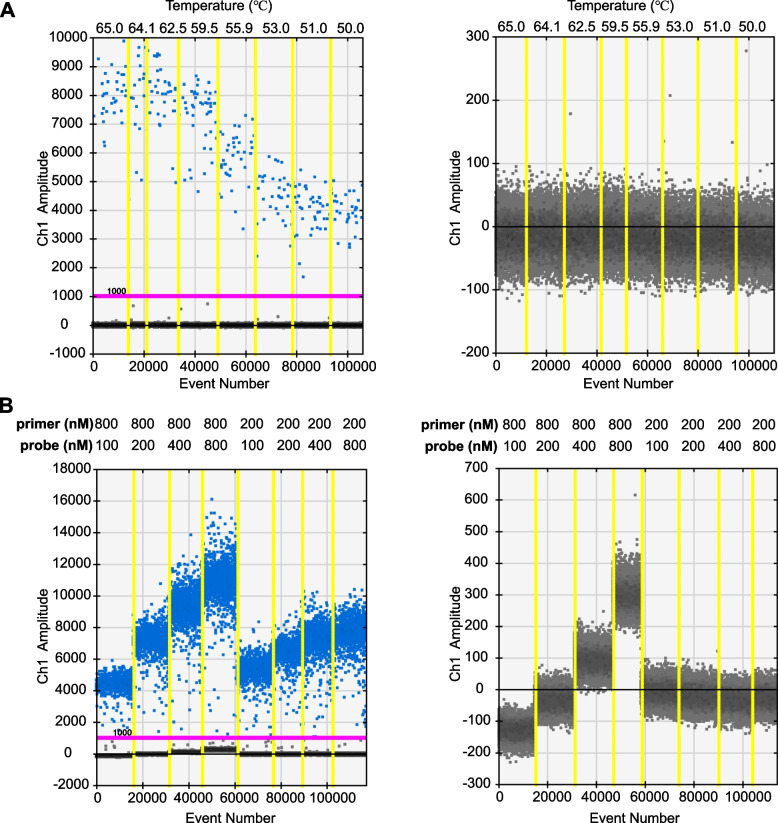
Optimization of the ddPCR system. **A** Optimization of annealing temperature. The plasmid based standard DNA template (left panel) or enzyme-free water (right panel) were used for amplification. A set of gradient temperatures were labeled on the top of figures. Eight reactions are separated by yellow lines, the amplitude of fluorescent readouts, and positive (blue) and negative (gray) droplets are separated by the threshold (pink line). **B** Optimization of the concentration of primers and probe. The standard DNA template (left panel) or enzyme-free water (right panel) were used for amplification. A series of different combinations of primers and probe were labeled on the top of figures

To establish a ddPCR system for detecting the *CCDC6*::*RET* fusion, we constructed a plasmid containing *CCDC6*::*RET* fusion sequence and linearized it for using as the standard template, and the annealing temperature and concentration were optimized. Specifically, the ideal annealing temperature was determined by gradient PCR. As shown in Fig. 2A, as the temperature increased from 50 °C to 62.5 °C, the fluorescence of positive droplets gradually increased and showed better separation for positive and negative droplets, while the efficiency was no longer increased when the temperature exceeded 62.5 °C. Therefore, the ideal annealing temperature was set as 62.5 °C. Next, we explored the ideal concentrations for primer and probe by testing a series of concentration combinations. Compared to 200 nM of primers, 800 nM showed more fluorescence; when the primer concentration was 800 nM, probe concentration at 200 nM showed best performance with respect to positive and negative droplets separation (Fig. 2B). A primer concentration at 800 nM and a probe concentration at 200 nM were chosen for further experiments.

#### Comparison of ddPCR with qRT-PCR for RET fusion detection

**Fig. 3:**
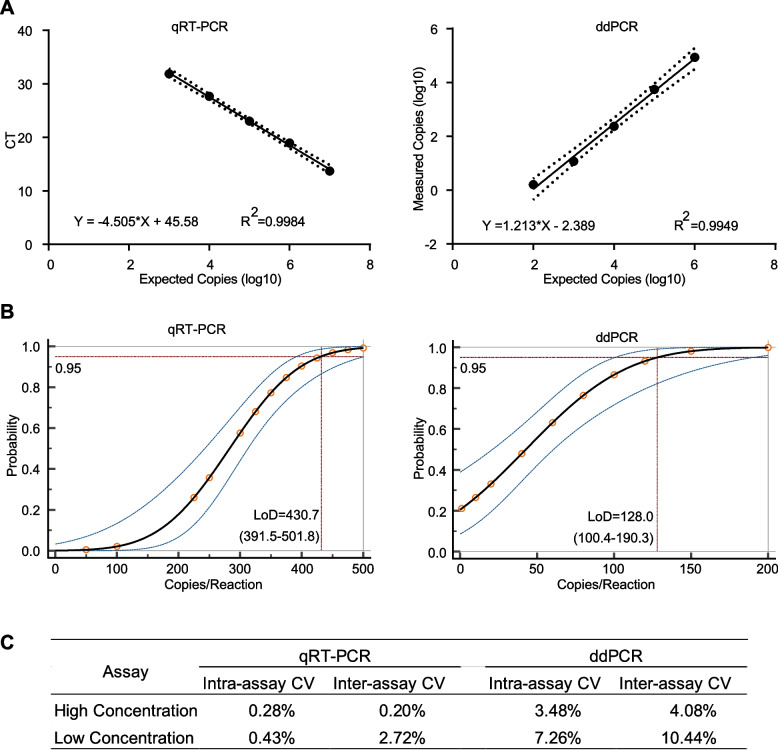
Analytical performance of qRT-PCR and ddPCR for *CCDC6*::*RET* fusion detection. **A** Sensitivity of qRT-PCR and ddPCR assays. Measured values were plotted versus expected copies of gene fusion from serial dilutions. The black line represented the linear regression curve, and the outer dished lines represented the 95% confidence intervals (CIs). **B** Limit of detection (LoD) analysis for qRT-PCR and ddPCR by probit analysis. X-axis represented the expected concentration (copies/reaction). Y-axis represented the fraction of positive results at a certain concentration. The black line represented the dose–response probit curve, and the outer lines indicated the 95% CIs. **C** Variation of qRT-PCR and ddPCR. High concentration: expected 5000 copies/reaction. Low concentration: expected 500 copies/reaction. Three replicates were set at each concentration for calculating the intra-assay coefficient of variation (CV), and three different time points for the inter-assay CV

Next, we compared the analytical performance of ddPCR with qRT-PCR. To start this, the linearity of qRT-PCR and ddPCR was assessed by quantifying serially ten-fold diluted standard templates. As a result, both qRT-PCR and ddPCR curves exhibited high linearity with a R^2^ of 0.998 and 0.995, respectively (Fig. 3A). To determine the limit of detection (LoD) of the two methods, DNA standard was diluted to a series of concentrations below the minimum detection range. Eight replicates were performed at each concentration. The LoD was analyzed by probit regression with a 95% probability. As shown in Fig. 3B, the LoD of qRT-PCR was 430.7 (95% CI: 391.5–501.8) copies/reaction while that was 128.0 (95% CI: 100.4–190.3) copies/reaction in ddPCR assay, suggesting ddPCR is more sensitive than qRT-PCR in samples with low copy of *CCDC6*::*RET* fusion. Based on the linear range and LoDs, we chose 5,000 copies/reaction as a high concentration and 500 copies/reaction as a low concentration to estimate the precisions of the two methods. The coefficient of variation (CV) values of the two methods was shown in Fig. 3C. The inter assay CV ranged from 4.1% to 10.4% and the intra assay CV ranged from 3.5% to 7.3% for ddPCR, and for qRT-PCR inter assay CV was from 0.2% to 2.7% and the intra assay CV ranged from 0.3% to 0.4%.

#### Detection of CCDC6::RET fusion in PTC samples

**Fig. 4:**
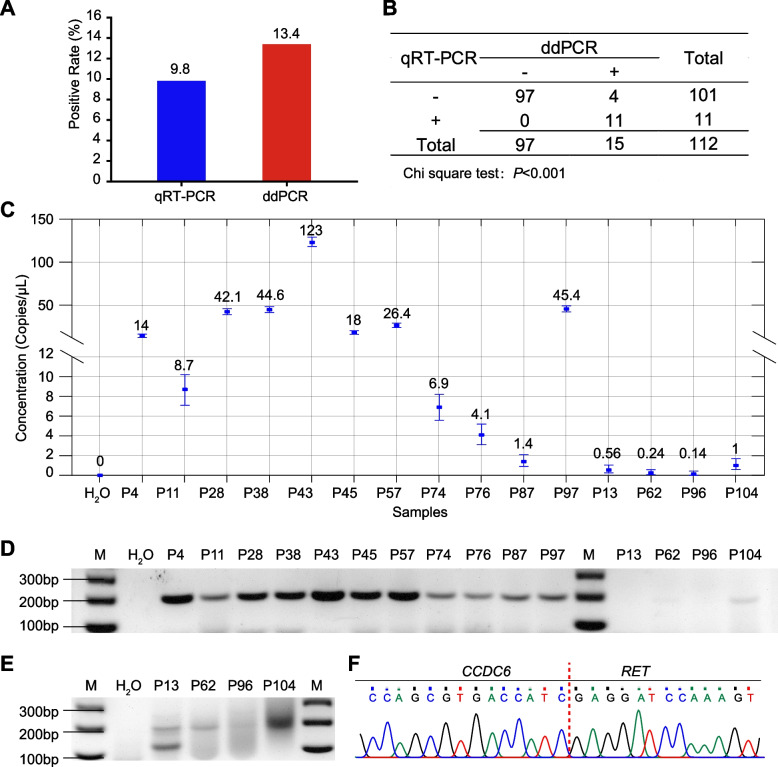
Detection of *CCDC6*::*RET* fusion in PTCs. **A** Frequency of *CCDC6*::*RET* fusion identified by qRT-PCR and ddPCR in 112 PTC samples. **B** Cross tabulation of the two methods. Chi-square test was used to evaluate effectiveness. **C** Concentrations of *CCDC6*::*RET* fusion positive samples identified by ddPCR. X-axis represented sample ID; Y-axis represented the concentration of RET fusion (copies/uL). **D** Agarose gel electropherograms of the PCR products of *CCDC6*::*RET* fusion positive samples. M, DNA size marker. **E** Amplification of the low copy *RET* fusion positive samples by nested PCR. **F** Representative electropherograms of the *CCDC6*::*RET* fusion

To assess the efficiency of ddPCR for *CCDC6*::*RET* fusion detection in clinical samples, we applied ddPCR and qRT-PCR in 112 patients with PTC and compared results from the two methods. Eleven (9.8%) *RET* fusion-positive samples were detected by qRT-PCR, while the number of positive cases increased to 15 (13.4%) when the ddPCR was performed (Fig. 4A). Notably, all the 11 positive samples identified by qRT-PCR could be recognized by ddPCR, and 4 additional positive samples were identified by ddPCR, but not by qRT-PCR (Fig. 4B). Actually, all samples with > 1 copy/uL of *CCDC6*::*RET* fusion were detectable by qRT-PCR (Fig. 4C), and they were clearly visualized by RT-PCR (Fig. 4D, S1A). The four cases with a concentration of 1 copy/uL or below can be detected by nested PCR except for one sample that had an extremely low concentration of *RET* fusion (Fig. 4E, S1B). All the 14 visualized samples from RT-PCR were confirmed by Sanger sequencing (Fig. 4F). These data suggested that ddPCR had a better capability for *CCDC6*::*RET* fusion detection than qRT-PCR.

## Discussion

*RET* fusion is a one of common genetic drivers in multiple human cancers, including PTC. Fluorescence in situ hybridization (FISH), qRT-PCR, and next-generation sequencing (NGS) were currently used for detecting *RET* fusions. Although FISH is considered as the gold standard for fusion detection, it is time-consuming and requires experienced personnel [26, 27]. Similarly, NGS is labor-intensive, time-consuming and expensive although it is one of the most comprehensive and sensitive methods for genetic analysis. The easy accessibility and high sensitivity of ddPCR makes it became a new trend for detecting specific genetic alteration [28, 29]. In this study we developed a ddPCR method for detection of *CCDC6*::*RET* fusion, the most frequent subtype of *RET* fusions. By optimizing the primer and probe concentrations and annealing temperature, the ideal condition for *CCDC6*::*RET* fusion detection was established.

Compared with the widely used qRT-PCR method, the LoD of our method is remarkably low, suggesting the sensitivity of this new method is superior to qRT-PCR. In support of this, when we applied these two methods in 112 PTC samples, all the fusion-positive cases identified by qRT-PCR were detectable in the ddPCR system, and ddPCR identified 4 additional *CCDC6*::*RET* fusion-positive samples. Importantly, although the copy number of *CCDC6*::*RET* is very low in the 4 samples, the fusion were successfully confirmed by Sanger sequencing except for the sample with the lowest copy number. This phenomenon is consistent with previous findings that ddPCR is more sensitive than Sanger sequencing for the detection of driver mutations [30, 31], although we cannot exclude the possibility that the unconfirmed positive case was a false-positive result from ddPCR.

The frequency of RET fusion detected by qRT-PCR in the current study was in accordance with previous findings that the *RET* fusion frequency was about 4–9% in sporadic PTC [6, 32]. However, the ddPCR assay showed that the fusion frequency increased to 13.4%, suggesting that the incidence of *RET* fusion in PTC might be underestimated. By analyzing the sequencing data of PTC from the TCGA cohort, we found that *RET* fusions were mutually exclusive with somatic genetic alterations in *BRAF*, *RAS*, *EIF1AX* and *TERT* except in one sample, further indicating an oncogenic role of *RET* fusion in PTC tumorigenesis. Moreover, although the relationship between *RET* fusion and clinical behavior and outcome of PTC is controversial [11, 33, 34], recent studies involving large sample numbers showed that *RET* fusions were associated with more aggressive characteristics of PTC, including extrathyroidal extension, lymph node and distant metastases, radioiodine refractory, and worse prognosis [12, 35, 36].

Advanced patients with *RET* fusions can benefit from targeted therapy [15]. A recent clinical trial showed that 79% of patients with previously treated *RET* fusion- positive thyroid cancer had a response to *RET* kinase specific inhibitor selpercatinib [17]. Since the ddPCR system established in this study provides a sensitive method for *RET* fusion detection, it would be definitely benefits more thyroid cancer patients in the clinic. In addition to *RET* fusion-positive thyroid cancers, selpercatinib was also demonstrated durable and robust responses in *RET* fusion-positive NSCLC and 12 other types of solid tumor [18, 37, 38]. Since the ddPCR system established in this study provided a sensitive method for *CCDC6*::*RET* fusion detection, application of this method to these cancer types would be benefits more *RET* fusion-positive patients in the clinic. It should be noted that the method reported here is designed for *CCDC6*::*RET*, but not for other subtypes of RET fusions, therefore multiplex ddPCR system for detecting all subtypes of RET fusion is needed to be established.

## Conclusions

This study has developed a highly sensitive and accurate method for *CCDC6*::*RET* fusion detection by ddPCR. It is more sensitive than qRT-PCR and has the potential to become a reliable alternative technique to determine the presence of *CCDC6*::*RET* fusion in patients with PTC.

## Supplementary Information

**Additional file 1.**

## Data Availability

The datasets used and/or analyzed during the current study are available from the corresponding author on reasonable request.

## Abbreviations

PTC: Papillary thyroid cancer
PDTC: Papillary differentiated thyroid cancer
ATC: Anaplastic thyroid cancer
MTC: Medullary thyroid cancer
FTC: Follicular thyroid cancer
ddPCR: Droplet digital PCR
qRT-PCR: Quantitative reverse transcription PCR
RET: Rearranged during Transfection
CCDC6: Coiled-coil domain containing 6
NCOA4: Nuclear receptor co-activator 4
