# Supplementary figures and images for: Highly sensitive droplet digital PCR for detection of RET fusion in papillary thyroid cancer
Source: BMC Cancer. 2023 Apr 20;23:363. doi: 10.1186/s12885-023-10852-z (PMC10120194; doi:10.1186/s12885-023-10852-z)

**A**

Uncropped gel for Figure 4 D

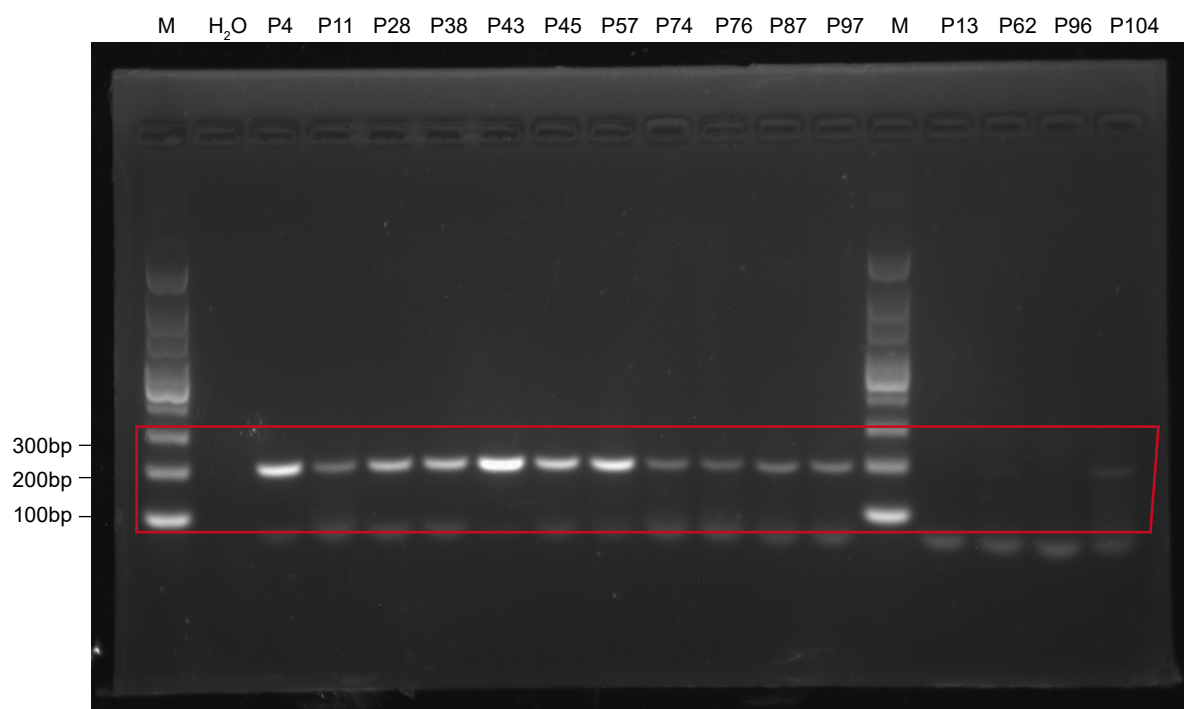**B**

Uncropped gel for Figure 4 E

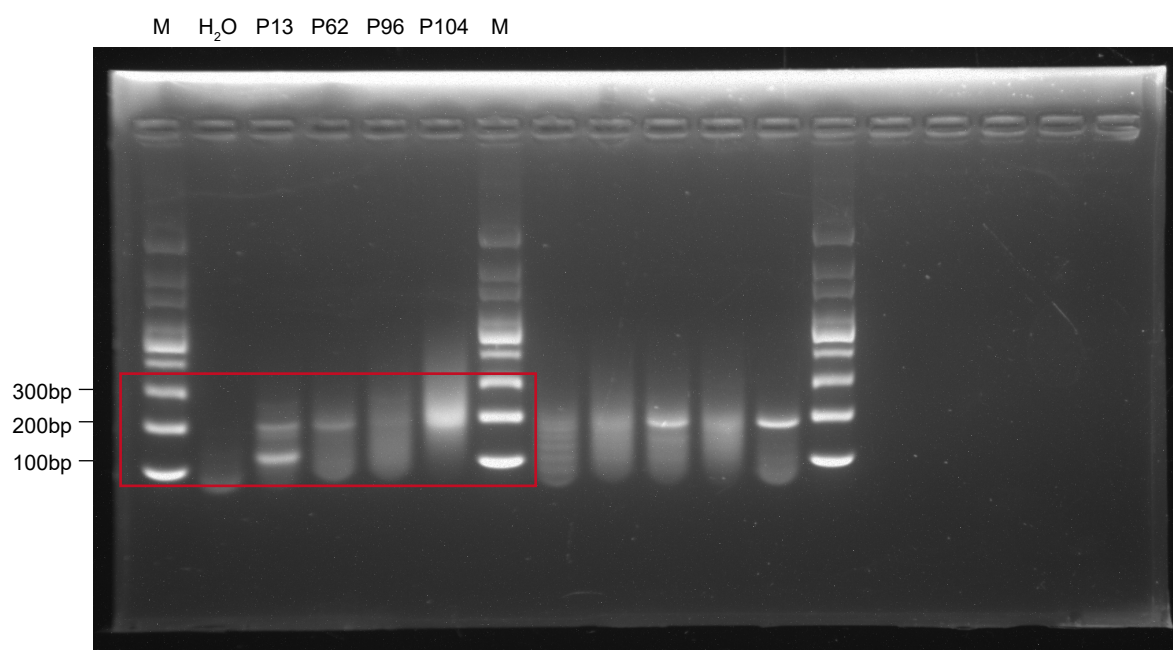

Supplement: Supplementary file 1 — Additional file 1. [file 12885_2023_10852_MOESM1_ESM.pdf]
